# Acute stress does not affect risky monetary decision-making

**DOI:** 10.1016/j.ynstr.2016.10.003

**Published:** 2016-11-02

**Authors:** Peter Sokol-Hessner, Candace M. Raio, Sarah P. Gottesman, Sandra F. Lackovic, Elizabeth A. Phelps

**Affiliations:** a Department of Psychology, New York University, 4-6 Washington Place, New York, NY 10003, United States; b Center for Neural Science, New York University, 4-6 Washington Place, New York, NY 10003, United States

**Keywords:** Risk attitudes, Stress, Cortisol, Decision-making, Loss aversion, HPA, Hypothalamic-Pituitary-Adrenal, CPT, Cold Pressor Test, CI, Confidence Interval

## Abstract

The ubiquitous and intense nature of stress responses necessitate that we understand how they affect decision-making. Despite a number of studies examining risky decision-making under stress, it is as yet unclear whether and in what way stress alters the underlying processes that shape our choices. This is in part because previous studies have not separated and quantified dissociable valuation and decision-making processes that can affect choices of risky options, including risk attitudes, loss aversion, and choice consistency, among others. Here, in a large, fully-crossed two-day within-subjects design, we examined how acute stress alters risky decision-making. On each day, 120 participants completed either the cold pressor test or a control manipulation with equal probability, followed by a risky decision-making task. Stress responses were assessed with salivary cortisol. We fit an econometric model to choices that dissociated risk attitudes, loss aversion, and choice consistency using hierarchical Bayesian techniques to both pool data and allow heterogeneity in decision-making. Acute stress was found to have no effect on risk attitudes, loss aversion, or choice consistency, though participants did become more loss averse and more consistent on the second day relative to the first. In the context of an inconsistent previous literature on risk and acute stress, our findings provide strong and specific evidence that acute stress does not affect risk attitudes, loss aversion, or consistency in risky monetary decision-making.

## 1 Introduction

Because risky decisions are both ubiquitous and must often be made under stress, it is imperative to understand the interactions between stress and choices under risk. However, despite a number of studies examining acute stress and risky monetary decision-making (see Table S1), it is as yet unclear whether and how they interact. In the gain domain, several studies find evidence for more gambling^1^ under acute stress (i.e. riskier choices; less risk aversion; more utility function convexity) (Preston et al., 2007, Starcke et al., 2008, Putman et al., 2010, Pabst et al., 2013b, Pabst et al., 2013c), while others find *less* gambling under stress (i.e. safer choices; more risk aversion; more utility function concavity) (Porcelli and Delgado, 2009, Cingl and Cahlikova, 2013), no changes in gambling (von Dawans et al., 2012, Delaney et al., 2014, Kandasamy et al., 2014), or both more and less gambling depending on factors like gender (Lighthall et al., 2009, van den Bos et al., 2009), time (Pabst et al., 2013a), trait anxiety and depressive symptoms (Robinson et al., 2015), or outcome magnitude (von Helversen and Rieskamp, 2013). Even with respect to gender, the findings are equivocal: roughly equal numbers of studies found interactions with gender (Preston et al., 2007, Lighthall et al., 2009, van den Bos et al., 2009) as did not (Starcke et al., 2008, Pabst et al., 2013b, von Helversen and Rieskamp, 2013, Kandasamy et al., 2014).

One reason for this apparent inconsistency may be that, with one exception (Kandasamy et al., 2014; see Table S1), all the studies mentioned above used the same problematic measure of risky decision-making: a simple probability of gambling. This coarse measure is inadequate because choices between more and less risky options reflect the combined contributions of multiple different processes. For example, someone under stress might gamble less (that is, their probability of gambling might go down) because they dislike the element of chance or risk in the gamble (termed risk attitudes), because they overweight the risky loss relative to the risky gain (termed loss aversion), or simply because they are choosing more (or less) consistently than before despite having the same risk attitudes and loss aversion. Depending on the kinds of choices, other factors can also influence the probability of gambling, including probability weighting (the subjective, as opposed to objective, probability of an event occurring), ambiguity aversion (the distaste for unknown probabilities in decision options), or even dynamic updating when learning in complex, changing, or experiential settings.

Concluding that changes in the probability of gambling are due to changes in attitudes toward risk without dissociating other relevant processes would be analogous to concluding that stress affects memory recall after a study in which participants memorized items and performed a recognition test all while under stress. Such a conclusion would be obviously flawed as differences in recognition could reflect changes in perception, encoding, consolidation, familiarity, or recall – and without careful design and analysis, would all be thoroughly confounded. By the same token, the fundamentally different processes underlying risky choices must be simultaneously and separately quantified, or otherwise accounted for, in order to understand the ways in which acute stress does and does not affect decisions under risk.

In this study, we sought to dissociate and quantify three separable decision-making processes under acute stress in a fully-crossed within-subjects design. Briefly, participants came in on each of two days, identical except for experiencing an acute stress or control manipulation with equal probability on each day. Individual differences in HPA axis activity were objectively quantified with four measurements of salivary cortisol per day (Velasco et al., 1997, McRae et al., 2006). Participants' decision-making was also quantified with a risky decision-making task (Sokol-Hessner et al., 2009, Sokol-Hessner et al., 2013, Sokol-Hessner et al., 2015, Sokol-Hessner et al., 2015) that, in combination with an econometric model of valuation and decision-making, allowed the separation of risk attitudes, loss aversion, and consistency in decision-making for each participant on each day. Finally, statistically powerful hierarchical Bayesian analysis methods were used to pool the data from 120 participants, both leveraging individual differences and group-level analysis to identify how acute stress affects or spares the three measured processes contributing to risky decision-making.

## 2 Methods

### 2.1 Participants

A total of 122 participants completed the task. Two participants were subsequently dropped when it became apparent that they did not understand the mechanics of the task, leaving a total of 120 participants (64 female; mean age = 22.4, standard deviation = 4.5). Our fully crossed design (Stress or Control condition on each of Day 1 and Day 2) resulted in four groups (Stress-Stress, Stress-Control, Control-Stress, or Control-Control). Participants were evenly distributed (N = 30) across these four groups. One participant was excluded from cortisol analyses as their mean salivary cortisol level was more than thirty standard deviations above the group mean.

All participants provided informed consent in accordance with procedures approved by NYU's University Committee on Activities Involving Human Subjects.

### 2.2 Study design

#### 2.2.1 Overall study design

**Fig. 1:**
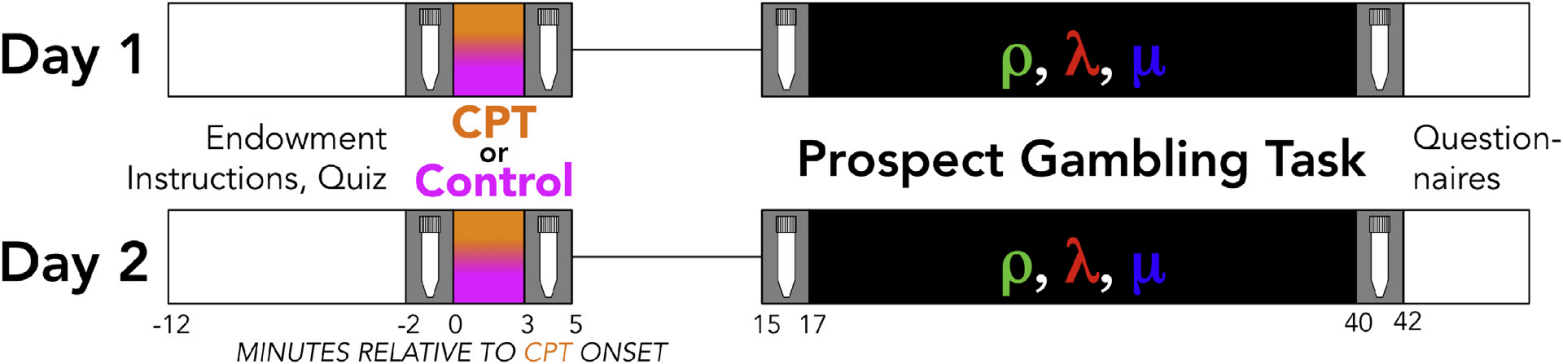
Study procedure. Participants came into the lab for two sessions, a minimum of 2 days apart at roughly the same time of day (see Methods). The first day began with monetary endowment, task instructions, and a basic comprehension quiz before the first (baseline) cortisol sample was taken (represented in the figure by a schematic salivary collection tube). After either undergoing the cold pressor test (CPT) or the lukewarm water control, participants gave a second salivary sample, waited 10 min for salivary cortisol levels to rise, and gave a third (pre-task) salivary sample. Participants then completed the risky decision-making task allowing estimation of risk attitudes (ρ, in green), loss aversion (λ, in red), and choice consistency (μ, in blue), after which they gave the fourth and final salivary sample, and completed a few basic debriefing questionnaires assessing their experience. The second day was identical to the first, except participants had an equal and independent chance of performing the CPT or lukewarm water control on each day. (For interpretation of the references to colour in this figure legend, the reader is referred to the web version of this article.)

All participants came in for two nearly identical sessions, separated by a mean of 5.3 days (standard deviation = 2.7; see Fig. 1; delay between sessions did not differ as a function of Group: F(3,119) = 1.48, p = 0.22). All sessions began between 11:30a.m. and 5:20p.m. (Day 1 mean = 2:17p.m., standard deviation = 1.6 h; Day 2 mean = 2:12p.m., standard deviation = 1.5 h). Following consent, participants were immediately endowed with $30 and told they would be paid the outcome of a subset of the trials in the decision-making task. The experimenter then read the task instructions out loud as the participant silently read along, after which participants completed a brief comprehension quiz on task details, and completed practice trials under experimenter supervision.

The first of four saliva samples was then taken (see below), after which participants underwent either the cold pressor test (CPT; a common acute stress induction procedure; Velasco et al., 1997, McRae et al., 2006) or a lukewarm water control. In the CPT, participants submerge their non-dominant arm up to and including their elbow in 0–4 °C water for three minutes. The participant is asked to not speak during the CPT, and the time elapsed is not shared with the participant. The lukewarm water control used 30–32 °C water. Participants had an equal chance of undergoing the CPT or control condition on each of the two days. Immediately following the conclusion of the CPT (or control), a second saliva sample was collected, and then participants were given an 8-min break during which they were asked to sit quietly without using any digital devices. They then gave a third saliva sample, after which they completed the risky decision-making task which took roughly 23 min (see below; Sokol-Hessner et al., 2009, Sokol-Hessner et al., 2013, Sokol-Hessner et al., 2015, Sokol-Hessner et al., 2015). Finally, participants gave a fourth saliva sample and completed a post-study questionnaire.

Participants were paid $15 per hour, plus their adjusted $30 endowment at the end of each day. Fifteen trials were selected at random from the task and their outcomes summed with the endowment to produce the adjusted endowment. The mean adjusted endowment at the end of Day 1 was $53.08 (standard deviation = $22.08), and Day 2 was $51.80 (standard deviation = $18.19). The difference in payment between days was not significant (paired samples *t*-test, p = 0.62).

#### 2.2.2 Risky decision-making task

The main task of interest was a risky monetary decision-making task. As the task we used has been described in detail elsewhere (Sokol-Hessner et al., 2009, Sokol-Hessner et al., 2013), we will briefly summarize it here. Participants made 150 decisions between risky binary gambles and guaranteed alternatives. For 120 of the trials, termed “gain-loss trials”, the risky gamble consisted of equal chances of winning some amount or losing a different amount (amounts varied trial-to-trial), versus a guaranteed alternative of zero dollars. In the remaining thirty “gain-only trials”, the risky gamble yielded a positive amount or zero dollars with equal probability, and the guaranteed alternative was a smaller positive amount. The values used on each trial were unique (i.e. no trials were repeated). Trial order was random. The 50/50 probabilities used throughout the task effectively eliminated possible roles for ambiguity and probability weighting in the task, as all probabilities were explicitly known, and probabilities did not vary.

On each trial, the choice options were initially presented for 2s. After two seconds had passed, a response prompt (“?”) appeared prompting participants to enter their choice within two seconds. This was followed by an inter-stimulus interval (1s), the display of the outcome (either the outcome of the gamble or the guaranteed alternative depending on the participant's choice; 1s), and an inter-trial interval (1, 2, or 3s, uniformly distributed) before the next trial began.

The task had no temporal component to eliminate temporal discounting, included only two simple probabilities (0.5 and 1) to minimize the effect of any probability weighting, and was thoroughly instructed in detail and practiced to minimize learning and eliminate ambiguity.

#### 2.2.3 Cortisol measurement

Salivary samples were collected four times each day for each participant (see Fig. 1). For each sample, participants held a sterile synthetic polymer-based oral salivette under their tongue for two minutes, after which the swab was placed in a sterile collection tube and frozen at −20 °C. Frozen salivary samples were analyzed by Salimetrics Testing Services (a Clinical Laboratory Improvement Amendments-certified lab; Carlsbad, CA) using high-sensitivity enzyme immunoassay kits to assay cortisol levels.

### 2.3 Analysis

#### 2.3.1 Cortisol analysis

An initial visual inspection of raw cortisol values identified one participant (mentioned in Section 2.1, Participants) with a mean salivary cortisol level more than thirty standard deviations above the group mean. This participant was removed from any subsequent cortisol analyses.

To confirm the efficacy of the CPT, we first analyzed the change in cortisol measurement from the baseline cortisol sample taken immediately before the CPT to the three later time points (immediately after the CPT, prior to the decision-making task, and immediately after the task). The Stress condition resulted in significantly larger increases in cortisol relative to the Control condition across both the pre- and post-task time points (see Supplemental Materials for tests within each group, across days and timepoints). To quantify individual differences in cortisol reactivity for use as a covariate in behavioral analyses, we focused on the change in cortisol between the baseline (1) and pre-task (3) time points. Because raw cortisol change values were positively skewed, but spanned zero, we used a modified log procedure similar to that used elsewhere (e.g. Otto et al., 2013) to reduce skewness while maintaining the meaningfulness of zero values (ΔCortisol = log([Cortisol_3_-Cortisol_1_]+0.5)-log(0.5)).

#### 2.3.2 Behavioral analysis

Behavioral analysis proceeded in two main portions, the first of which consisted of examining changes in the simple probability of choosing the risky gamble across days as a function of condition (Stress vs. Control), replicating the analysis approach used in many other studies of risky decision-making under stress (see Table S1).

For the second main analysis, we fit prospect theory-inspired models of the non-linear processes underlying valuation and choice using a hierarchical Bayesian approach. The basic model was identical to that used previously (see Equations (1), (2), (3); Sokol-Hessner et al., 2009, Sokol-Hessner et al., 2013, Sokol-Hessner et al., 2015, Sokol-Hessner et al., 2015).

(1) $$u\left(x^{+}\right)=p\left(x\right)\times x^{\rho }$$

(2) $$u\left(x^{-}\right)=p\left(x\right)\times -\lambda \times {\left(-x\right)}^{\rho }$$

(3) $$p\left(choose\;gamble\right)={\left(1+e^{-\mu \times \left(u\left(gamble\right)-u\left(guaranteed\right)\right)}\right)}^{-1}$$

Equations (1), (2) determine the utility (*u(x)*) of the objective monetary amounts in the risky gamble and the guaranteed alternative. The difference in utility between the gamble and the guaranteed alternative is then used to calculate the probability of choosing the gamble as given by the standard softmax function in Equation (3). The model fits three parameters describing three distinct aspects of participants' decision-making. The parameter ρ (rho), captures risk attitudes or the diminishing marginal utility of money (represented by the curvature of the utility function), and is constrained to be the same across the gain and loss domains. When ρ = 1, participants are risk neutral; less than 1 indicates risk aversion for gains and risk seeking for losses, and greater than 1 indicates risk seeking for gains and risk aversion for losses. The parameter λ (lambda) quantifies loss aversion defined as the relative multiplicative weighting of losses to gains in choices. A λ of 1 indicates gain-loss neutrality (i.e. similar weight), while values greater than 1 indicate loss aversion, and less than 1 indicate gain-seeking. Finally, μ (mu) quantifies participants' internal consistency in choices. Higher values of μ represent greater consistency across decisions, versus lower values which indicate noisiness in decision-making. Critically, the inclusion of a number of both gain-loss and gain-only trial types in the task (see Section 2.2.2 Risky Decision-Making Task) allowed the separation of these three free parameters.

The hierarchical Bayesian approach to fitting this model gave us a statistical advantage by explicitly modeling and fitting parameters at the level of the participant (e.g. participant 1's risk attitude) as well as at the level of the group (e.g. the mean population risk attitude). Using such a model, and therefore fitting all participants' data simultaneously, has the effect of reducing the influence of outliers or noise, and thus maximizing ‘signal’. It also has the benefit of allowing us to directly model the effect of interest – that is, the effect of acute stress on each of the three valuation and decision processes at both the population and individual participant levels.

Formally, we fit two main models: Model 1 took a “condition” approach (i.e. Stress/Control as a binary variable), while Model 2 took a “covariate” approach (i.e. the continuous effect of ΔCortisol).

(4) $$\theta_{i,j}=e^{\theta_{i}+Stress_{j}\times \Delta \theta_{Si}+Day_{j}\times \Delta \theta_{Di}}$$

(5) $$\theta_{i}∼Normal\left(\theta_{M},\theta_{V}\right)$$

(6) $$\text{Δ}\theta_{Si}∼Normal\left(\text{Δ}\theta_{SM},\text{Δ}\theta_{SV}\right)$$

(7) $$\text{Δ}\theta_{Di}∼Normal\left(\text{Δ}\theta_{DM},\text{Δ}\theta_{DV}\right)$$

Equation (4) describes how each parameter (ρ, λ, and μ; any one of which is represented here by θ) was modeled for participant *i* on day *j*. θ_i_ is participant i's baseline parameter value, Stress_j_ is a binary indicator for whether day j occurred in the Stress (1) or Control (0) condition, Δθ_Si_ is the parameter capturing the change in parameter θ due to Stress (“S”) for participant i, Day_j_ is a binary indicator for whether day j is Day 1 (0) or Day 2 (1), and Δθ_Di_ captures the change in parameter θ due to Day (“D”; e.g. repeated performance) for participant i. The three components (individual baseline parameter value; effect of Stress; effect of Day) were summed within an exponential to prevent final parameter values (θ_i,j_) from being non-positive (zero is the lower bound for each of ρ, λ, and μ). Equations (5), (6), (7) illustrate how individual level parameters (e.g. θ_i_) were Gaussian-distributed around population means (e.g. θ_M_) and standard deviations (e.g. θ_V_).

Although Equation (4) is written for the “condition” approach (Model 1), the “covariate” approach (Model 2) is identical, with the exception of the Stress_j_ binary indicator being replaced by ΔCortisol_i,j_, representing the change in cortisol for participant i on day j (see Section 2.3.1 Cortisol Analysis).

These models were fit to the data using standard Markov-Chain Monte Carlo sampling methods in rStan (v2.2.0; Stan Development Team, 2015) as implemented in R (v3.0.2; R Core Team, 2015). For each of Model 1 and Model 2, 3000 samples were collected after a burn-in of 3000 samples (to allow chains to reach steady sampling states) on each of four chains, for a final total of 12,000 samples collected for each parameter (representing the posterior distribution over that parameter's possible values). For parameters of interest, 95% confidence intervals (CIs) were calculated using the samples, and examined to see if they contained zero (if they did not, we could be 95% confident that the true value of the relevant parameter was not zero). To calculate the magnitude of the effects of Stress (or ΔCortisol) and Day on the value function, parameter values were reconstructed with Equation (4), using mean sample values for the relevant parameters.

## 3 Results

### 3.1 Cortisol

Generally speaking, cortisol levels gradually decreased in the Control condition across the 2nd, 3rd, and 4th timepoints relative to the 1st (consistent with our afternoon testing time), and significantly increased in the Stress condition at the 3rd and 4th timepoints (pre- and post-risky decision-making task). For detailed comparisons as a function of timepoint, group, day, and condition, see Supplementary Materials and Fig. S1.

**Fig. 2:**
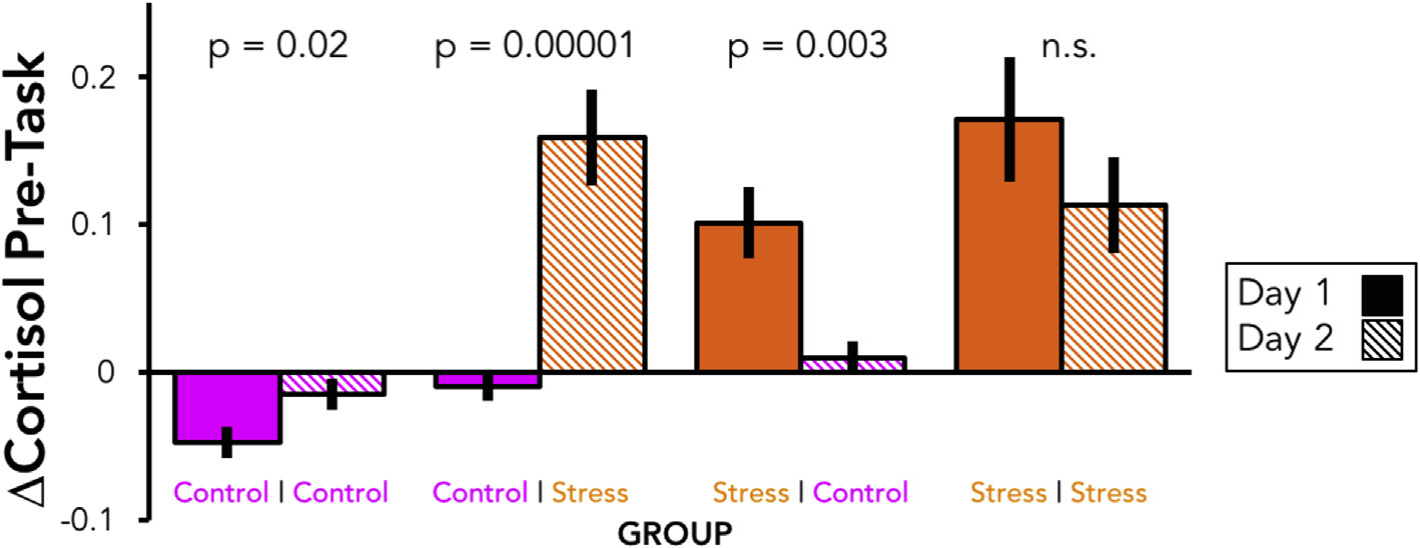
Change in cortisol at the third (pre-task) time point. Bars reflect the mean change in salivary cortisol (ug/ml) from the baseline sample to the pre-task sample. Orange bars indicate the stress condition, and purple bars the control condition, while solid bars indicate day 1, and striped bars indicate day 2. Bars are paired by participant group (each N = 30), and P values reflect paired t-tests between the change in cortisol values across days, within group. (For interpretation of the references to colour in this figure legend, the reader is referred to the web version of this article.)

Focusing on the change in cortisol at the 3rd timepoint (Fig. 2), we found large and significant differences between Day 1 and Day 2 using paired t-tests for the Control-Stress group (Day 1 = −0.01 μg/ml; Day 2 = 0.16 μg/ml; t(29) = 5.3, p = 0.00001), and the Stress-Control group (Day 1 = 0.10 μg/ml; Day 2 = 0.01 μg/ml; t(29) = 3.2, p = 0.003), and a small but significant difference in the Control-Control group (Day 1 = −0.05 μg/ml; Day 2 = −0.01 μg/ml; t(29) = 2.5 p = 0.02). The Stress-Stress group was not significantly different across days (Day 1 = 0.17 μg/ml; Day 2 = 0.11 μg/ml; t(28) = 1.7, p = 0.1). The modified log transformation used on these values to create the ΔCortisol variable used in covariate analyses (see below, Section 3.2.2) did not change the pattern of findings, as expected (paired t-tests on Day 1 vs. Day 2: Control-Control, p = 0.04; Control-Stress, p = 0.000008; Stress-Control, p = 0.002; Stress-Stress, p = 0.06).

We also performed a mixed-effects linear regression in R across all participants using the lmer package (Bates et al., 2015), predicting individuals' change in cortisol at the 3rd timepoint with a random intercept and fixed effects for Day, Stress, and a Day × Stress interaction. We found significant effects for the intercept (β = 0.06, p = 1.8 × 10^−8^) and Stress (β = 0.07, p = 8.9 × 10^−16^), but not Day (β = 0.01, p = 0.35), nor the Day x Stress interactive term (β = −0.01, p = 0.14), indicating that the CPT was effective in inducing increases in cortisol and that Day had neither simple nor interaction effects on cortisol.

### 3.2 Behavior

#### 3.2.1 Simple probability of gambling

Replicating previous analysis approaches (see Table S1), we examined the simple probability of choosing the gamble in our task. In paired t-tests within condition groups (e.g. Control-Control) comparing the probability of gambling across days, no group showed a significant change in gambling behavior (each N = 30; all p's > 0.18; see Fig. S2). Collapsing across the Stress-Control and Control-Stress groups (N = 60), paired t-tests revealed no significant difference in gambling under Stress versus Control (p = 0.80).

#### 3.2.2 Hierarchical behavioral models

##### 3.2.2.1 Model 1: stress/control condition

Estimates of the convergence of the chains on similar distributions of parameter samples (Rhat; when Rhat = 1, the model has converged and chains are very similar to each other; values above 1 suggest lack of convergence, i.e. chains that are very different from one another) indicated that the model fit well (mean Rhat for group-level parameters = 1.01).

First, we checked the baseline parameter estimates for risk attitudes, loss aversion, and consistency to ensure they replicated previous work (Tom et al., 2007, Sokol-Hessner et al., 2009, De Martino et al., 2010, Sokol-Hessner et al., 2013, Chumbley et al., 2014, Sokol-Hessner et al., 2015, Sokol-Hessner et al., 2015). Computing the mean sample values for each of the group-level baseline parameters and then using Equation (4) to transform those values to value function parameter space produced appropriate values (mean recovered ρ = 0.92, 95%CI = [0.85 0.97]; mean recovered λ = 2.22, 95% CI = [1.88 2.61]; and mean recovered μ = 25.9, 95% CI = [21.3 31.1]). These indicated participants were mildly risk averse for gains (risk seeking for losses), moderately loss averse, and somewhat consistent in their choices.

Examining the 95% CIs for the parameters capturing the change in each of ρ, λ, and μ due to Day (that is, the changes in each parameter on Day 2 relative to Day 1) illustrated that though there was no consistent change in risk attitudes (95% CI for Δρ_DM_ = [-0.05 0.03]), on Day 2 people became more loss averse (95% CI for Δλ_DM_ = [0.06 0.23]; mean recovered Day 2 λ = 2.57) and more consistent in their choices (95% CI for Δμ_DM_ = [0.15 0.39]; mean recovered Day 2 μ = 34.3).

**Fig. 3:**
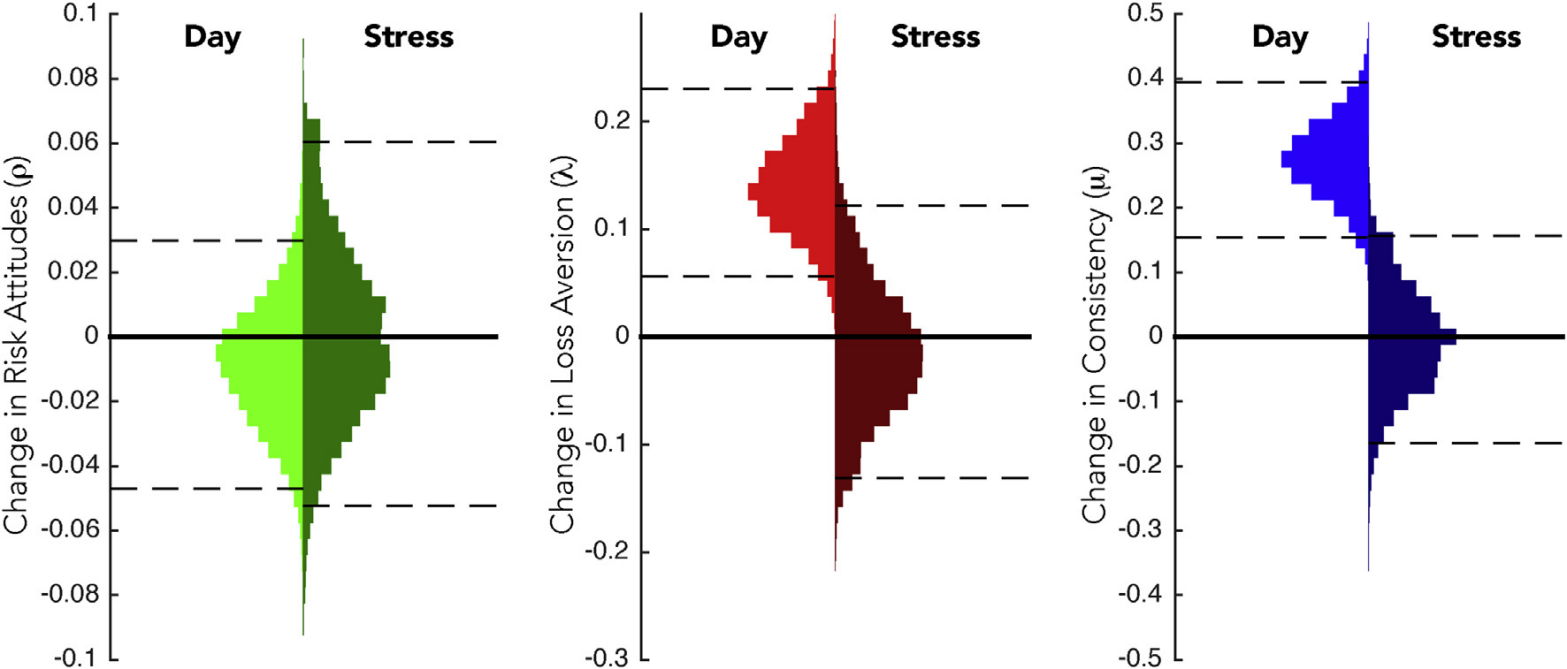
Changes in decision-making due to Day and Stress. Group mean changes in each of risk attitudes (ρ, green), loss aversion (λ, red), and consistency (μ, blue) due to repeated participation (“Day”) or the cold pressor test (“Stress”). Each histogram represents 12,000 samples from Model 1 (see Methods). 95% Confidence intervals are indicated for each histogram with dashed lines. Intervals excluded zero only for changes in loss aversion and consistency due to Day. (For interpretation of the references to colour in this figure legend, the reader is referred to the web version of this article.)

In contrast to the effects of Day, when examining the 95% CIs for the effects of the Stress condition, no consistent changes were found for any of the decision processes modeled (95% CI for Δρ_SM_ = [-0.05 0.06], mean recovered Stress ρ = 0.92; 95% CI for Δλ_SM_ = [-0.13 0.12], mean recovered Stress λ = 2.19; 95% CI for Δμ_SM_ = [-0.16 0.16], mean recovered stress μ = 25.8). As can be seen by a visual inspection of the 95% CIs, each is roughly centered on zero (see Fig. 3). These confidence intervals are small, as compared to the mean sampled standard deviations (e.g. θ_V_ from Equation (5)) for the group-level Gaussian distributions around which individual participants' overall mean parameter values are distributed (mean ρ_V_ = 0.24; mean λ_V_ = 0.84; mean μ_V_ = 0.87).

As the effects of stress inductions may be inconsistent in women (Kirschbaum et al., 1999, McCormick and Teillon, 2001, Andreano et al., 2008), we additionally ran Model 1 with only male participants (N = 56), generally replicating the findings of Model 1 when estimated for all participants. The mean recovered baseline parameters were comparable (mean recovered ρ = 0.90, 95%CI = [0.83 0.97]; mean recovered λ = 2.47, 95% CI = [1.79 3.25]; and mean recovered μ = 23.4, 95% CI = [16.9 32.2]). Examining the change due to Day replicated the null effect on risk attitudes (95% CI for Δρ_DM_ = [-0.03 0.06]) and the positive effect on consistency (95% CI for Δμ_DM_ = [0.30 0.70]), but the 95% confidence interval for the effect of Day on loss aversion no longer excluded zero (95% CI for Δλ_DM_ = [-0.07 0.28]; the CI had to be relaxed to 75.7% to exclude zero). The null effects of the Stress condition were replicated for all three value function parameters (95% CI for Δρ_SM_ = [-0.08 0.05]; 95% CI for Δλ_SM_ = [-0.22 0.24]; 95% CI for Δμ_SM_ = [-0.27 0.26]).

To test whether payment at the end of Day 1 altered decision-making on Day 2, we correlated the change in endowment at the end of Day 1 with the mean sample values of the change in ρ, λ, and μ for each participant. There was no significant correlation between the change in endowment and changes in risk attitudes (Δρ_Di_; r(118) = 0.06, p = 0.54) or consistency (Δμ_Di_; r(118) = 0.06, p = 0.49), but there was a correlation with the change in loss aversion (Δλ_Di_; r(118) = −0.32, p = 0.0004), such that small (or negative) changes to the endowment on Day 1 were correlated with more loss aversion on Day 2. The pattern and relative significance of the correlations held when using outlier-resistant non-parametric tests (e.g. Spearman's rho).

##### 3.2.2.2 Model 2: ΔCortisol

While Model 1 (binary Stress/Control coding) maximally leverages random experimental assignment, doing so ignores individual differences in responses to the stress manipulations. To address this issue, we fit Model 2, in which we included a ΔCortisol covariate (see Section 2.3.1) instead of the binary Stress/Control variable, to examine whether there was some more continuous relationship between cortisol levels and changes in risk attitudes, loss aversion, and/or consistency.

As with Model 1, Model 2 appeared to fit behavior well (mean Rhat for group-level parameters = 1.02), and replicated the mean baseline parameter estimates from Model 1 (mean recovered ρ = 0.92, 95%CI = [0.88 0.97]; mean recovered λ = 2.19, 95% CI = [1.87 2.57]; and mean recovered μ = 26.0, 95% CI = [21.7 30.4]).

Model 2 also replicated the finding that there was no consistent change in risk attitudes as a function of Day (95% CI for Δρ_DM_ = [-0.05 0.03]), but that participants were more loss averse and consistent on Day 2 relative to Day 1 (95% CI for Δλ_DM_ = [0.05 0.23], mean recovered Day 2 λ = 2.51; 95% CI for Δμ_DM_ = [0.18 0.40], mean recovered Day 2 μ = 34.6).

Finally, we found that there was no evidence for a continuous relationship between ΔCortisol and any of the value parameters (95% CI for Δρ_CM_ = [-0.17 0.09]; 95% CI for Δλ_CM_ = [-0.21 0.35]; 95% CI for Δμ_CM_ = [-0.27 0.43]; see Fig. S3 for histograms of sample distributions). It should be noted that because of the scaling of the ΔCortisol variable, these distributions additionally reflect very small effects if any. To illustrate this, we can use the mean ΔCortisol value from the Control condition (−0.04) and the Stress condition (0.20) to reconstruct the effect of cortisol on behavior (ΔCortisol = −0.04 vs. ΔCortisol = 0.20: ρ = 0.92 vs. 0.91; λ = 2.18 vs. 2.22; μ = 25.9 vs. 26.3).

As with Model 1, we additionally ran Model 2 on men only to check for gender specificity, generally replicating the findings from Model 2 estimated on all participants. Baseline parameter estimates were similar (mean recovered ρ = 0.89, 95%CI = [0.82 0.96]; mean recovered λ = 2.47, 95% CI = [1.82 3.28]; and mean recovered μ = 23.0, 95% CI = [16.6 30.6]). Like with Model 1's estimates from men only, we replicated the null effect of Day on risk attitudes (95% CI for Δρ_DM_ = [-0.02 0.07]) and the positive effect on consistency (95% CI for Δμ_DM_ = [0.31 0.70]), but did not replicate the effect of Day on loss aversion (95% CI for Δλ_DM_ = [-0.08 0.29]; the CI had to be relaxed to 71.5% to exclude zero), while ΔCortisol was found to have no consistent effect on any of the value parameters (95% CI for Δρ_CM_ = [-0.15 0.14]; 95% CI for Δλ_CM_ = [-0.60 0.56]; 95% CI for Δμ_CM_ = [-0.25 1.12]). See Fig. S3 for histograms of Model 2's parameter samples.

We ran additional models to test the sensitivity of these findings to the use of fixed instead of random effects, the use of more constrained models, and non-hierarchical maximum likelihood models. Findings of these ancillary models were identical to those above (see Supplementary Materials).

As with Model 1, the change in endowment at the end of Day 1 was significantly correlated with Model 2's Δλ_Di_ (r(117) = −0.31, p = 0.0007) but not with Δρ_Di_ (r(117) = 0.06, p = 0.53) or Δμ_Di_ (r(117) = 0.07, p = 0.47), findings that replicated with non-parametric Spearman's correlations.

#### 3.2.3 Basal cortisol and behavior

As some studies have found that baseline cortisol values may be related to risky decision-making (Chumbley et al., 2014), we tested whether basal cortisol values (i.e. the very first cortisol samples, taken prior to any CPT intervention) correlated with any of the individual parameter estimates from the hierarchical Bayesian analysis (see Section 3.2.2) for ρ, λ, and/or μ. Because estimates of behavior were calculated after the CPT, this analysis was limited to the 60 participants who were in the Control condition on Day 1 and whose behavior is most clearly at “baseline”. We used the parameter estimates calculated from Model 2, but findings were virtually identical using those from Model 1. Correlating the basal cortisol values on Day 1 with mean individual-level parameter samples on that day found no relationships with risk attitudes (Pearson's r(58) = 0.06, p = 0.65) nor loss aversion (Pearson's r(58) = −0.05, p = 0.68), and although there was a correlation with consistency (Pearson's r(58) = 0.35, p = 0.006), visual inspection suggested it was driven by outliers (Spearman's rho = 0.16, p = 0.23).

## 4 Discussion

Fully 78% of adults in the United States report experiencing stress at some point in the past month (APA, 2016), making it critical to understand whether and how intense and pervasive affective states like stress interact with decision-making. Here, we pursued this question using a large within-subjects design, an econometric model of valuation and decision-making that dissociates three underlying decision processes in risky decision-making, hierarchical Bayesian analysis that maximally combines data while allowing for heterogeneity in behavior, and objectively quantified endogenous acute stress responses. In doing so, we find no evidence for an effect of acute stress on risk attitudes, loss aversion, or consistency over choices.

We do find effects of repeated participation in the study, in that participants are more loss averse and more consistent on the second day relative to the first. A previous study from our lab also used a two-day design with the same task and although we observed increases in loss aversion on the second day, the increases were unrelated to Day 1 payment, and there were no changes in consistency (Sokol-Hessner et al., 2015b). Thus, while we encourage caution, especially in interpreting the effect of repetition on consistency, it does appear that participants weigh losses more heavily on their second day. One explanation could be that participants treated the money as “house money” (e.g. not their own) on the first day, despite our detailed instructions. When participants were paid real money at the end of the first day, they might have then returned on the second day, somehow more invested in the task, leading to greater loss aversion and consistency. However, while payment on Day 1 was correlated with the change in loss aversion, it did not correlate with changes in consistency. Additionally, this mechanism might also predict greater risk aversion for gains (risk seeking for losses), which we did not observe. As our study was not designed to test this hypothesis, we must rely upon future work for more definitive tests.

Though we find no effect of acute stress on risk attitudes (or loss aversion or consistency), what might explain previous findings to the contrary? First, it's possible that acute stress alters a decision-making process that we did not measure or manipulate in our study (e.g. probability weighting, temporal discounting, learning rates, ambiguity attitudes), but which was confounded with risky choices in other studies. As the vast majority of previous studies used the simple probability of gambling to assess risk attitudes (see Table S1), such conflation is very possible. For example, the Iowa Gambling Task and the Game of Dice Task are particularly popular paradigms, accounting for no fewer than seven of the previous studies on risky decision-making and stress, but their variable-probability, mixed gain & loss designs conflate many possible decision-making processes. Our task also had real monetary consequences and showed participants their outcomes on a trial-by-trial basis – hypothetical choices (or choices without feedback) may be differentially affected. Finally, it is of course possible that an overarching explanation for previously inconsistent findings may be relatively weak statistical power, either within the task (e.g. few trials) or at the study level (e.g. few participants; a brief review of the literature identifies a preponderance of low-power between-subjects designs, and an average of ∼60 participants/study; see Table S1).

More generally, this study examined decisions made over relatively simple explicitly described risky monetary options. To the extent to which decisions in other situations may involve other kinds of options, it is possible that stress may affect decision-making – but our findings suggest that such an effect would not be due to changes in risk attitudes, loss aversion, or choice consistency.

Additionally, while this study focused on acute stress, there is evidence that chronic, longer-term stress may alter decisions under risk. One study found that cortisol administration for eight consecutive days increased risk aversion (decreased gambling; Kandasamy et al., 2014), while another used hair samples to estimate approximate cortisol exposure over the previous two months, finding that chronic levels of cortisol were unrelated to risk attitudes but instead were negatively correlated with loss aversion (Chumbley et al., 2014).

The differences between endogenous and exogenous cortisol, acute and chronic stress levels, physiological and social stressors, cortisol and other biomarkers of stress, and other factors governing when, how, and in what context stress responses occur may ultimately prove critical to our understanding of the interactions between stress and decision-making. Nevertheless, the findings from our robust design and analysis combining for the first time quantitative estimation of risky decision-making and objective manipulations of acute stress, in the context of inconsistent previous findings, suggest that acute stress does not affect risk attitudes, loss aversion, or consistency in risky monetary decision-making.

## Footnotes

1 Risk attitudes are commonly represented by the curvature of the utility function (concave for gains, and convex for losses, a difference known as the reflection effect). This curvature leads to risk aversion for gains, and risk seeking for losses. More pronounced curvature entails more risk aversion for gains *and* more risk seeking for losses, while less curvature (more linearity) entails the opposite: less risk aversion for gains, and less risk seeking for losses. For legibility and for consistency with the extant literature, when we state “more gambling” and the like in this paper, we are referring to the gain domain unless specifically indicated otherwise, and we mean to imply the opposite for the loss domain.
