# Supplementary figures and images for: Acute stress does not affect risky monetary decision-making
Source: Neurobiol Stress. 2016 Nov 2;5:19–25. doi: 10.1016/j.ynstr.2016.10.003 (PMC5145911; doi:10.1016/j.ynstr.2016.10.003)

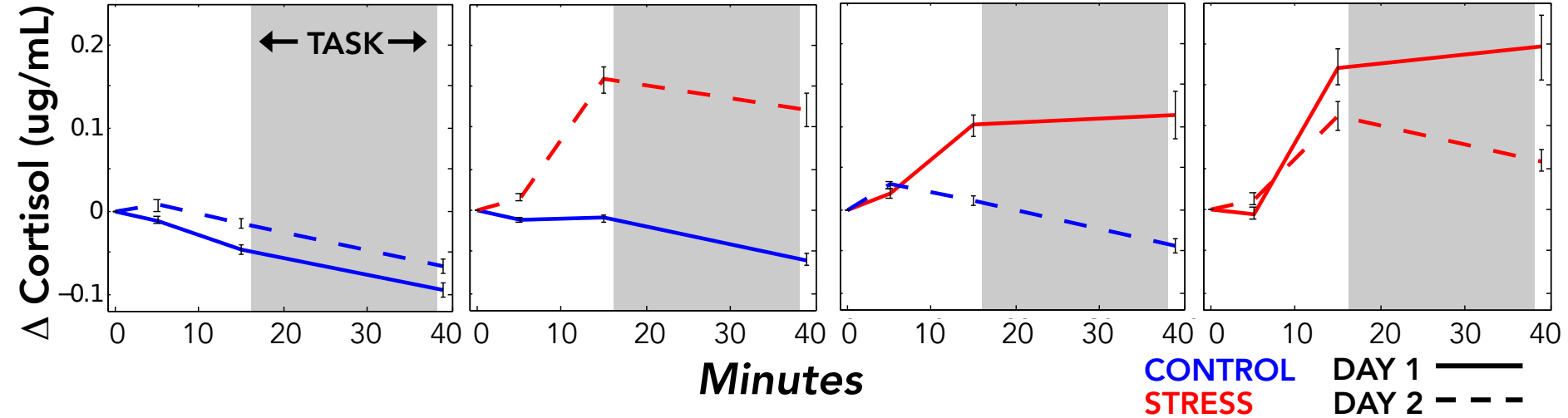

Supplement: Fig. S1 [file mmc1.pdf]

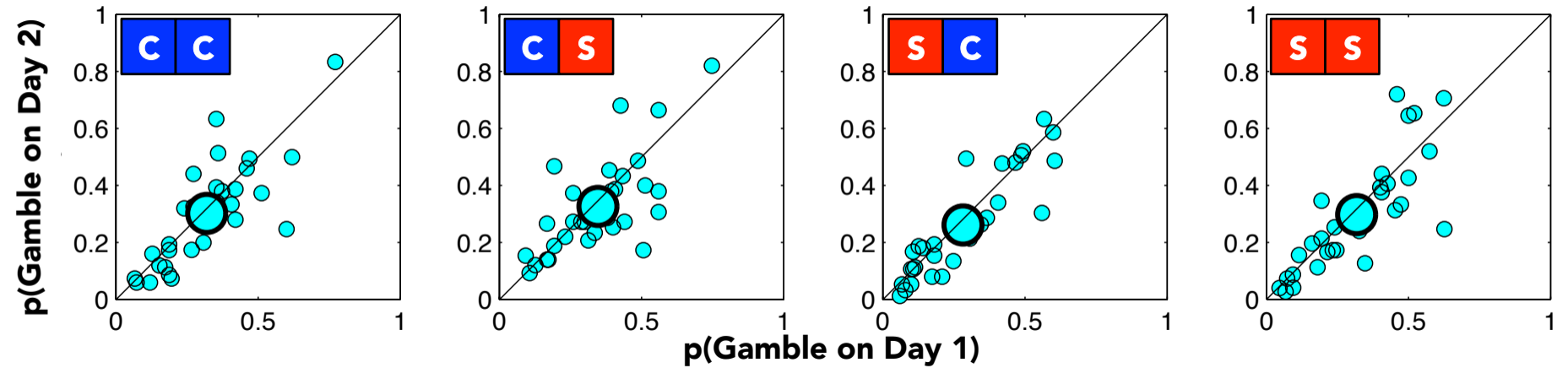

Supplement: Fig. S2 [file mmc2.pdf]
