# Supplementary material for: Acute stress does not affect risky monetary decision-making
Source: Neurobiol Stress. 2016 Nov 2;5:19–25. doi: 10.1016/j.ynstr.2016.10.003 (PMC5145911; doi:10.1016/j.ynstr.2016.10.003)
Supplement: Fig. S3 [file mmc3.pdf]

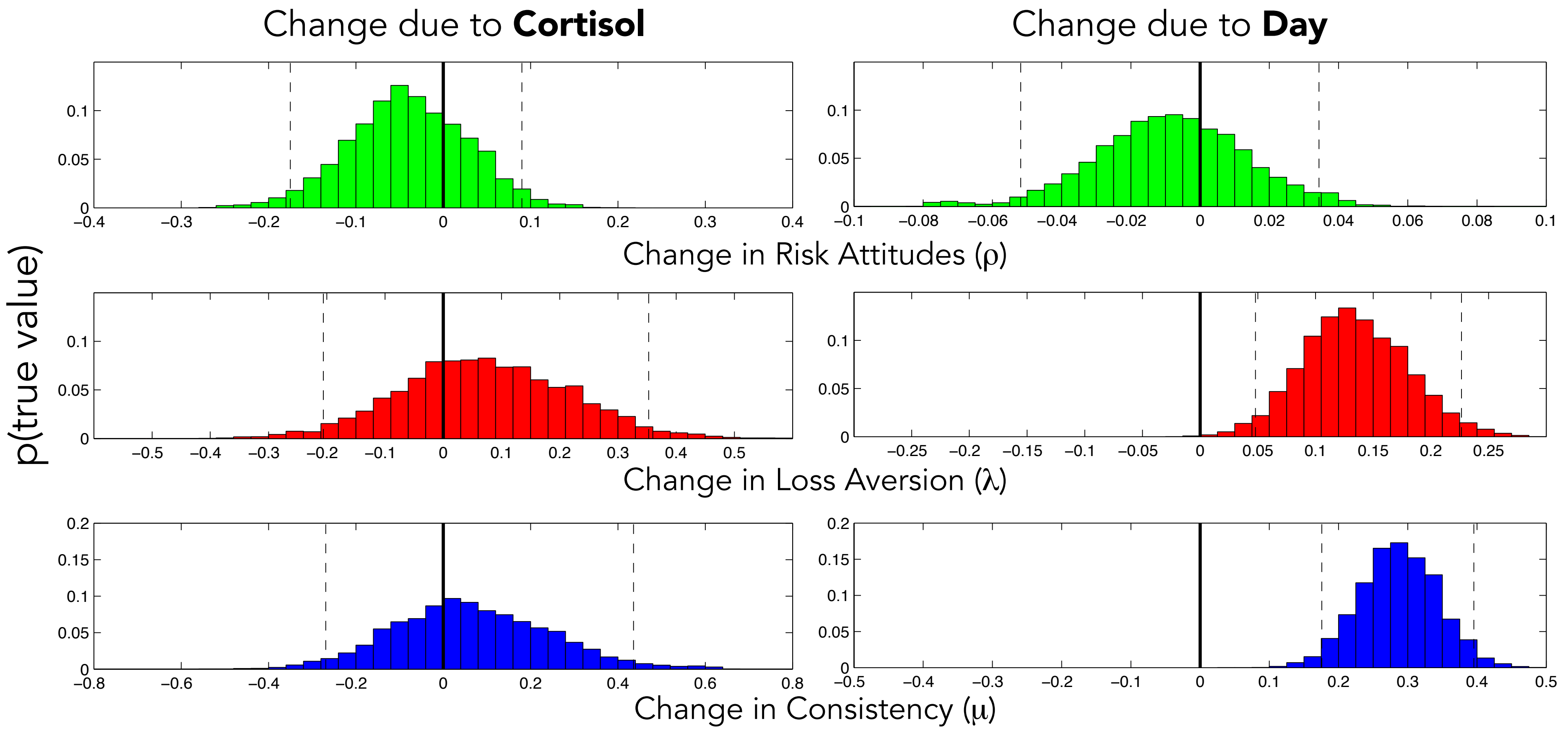

Change due to **Cortisol**
Change due to **Day**
p(true value)
Change in Risk Attitudes (ρ)
Change in Loss Aversion (λ)
Change in Consistency (μ)
