# Supplementary material for: Acute stress does not affect risky monetary decision-making
Source: Neurobiol Stress. 2016 Nov 2;5:19–25. doi: 10.1016/j.ynstr.2016.10.003 (PMC5145911; doi:10.1016/j.ynstr.2016.10.003)
Supplement: Table S1 [file mmc4.pdf]

1st Author	Year	Journal	Stressor	Task	Risk Measure	Other decision measures	Btwn/ Within?	N	Pwr	Rough finding	M/F?	G/L?	Notes	Implied Effect on ρ
Preston et al	2007	Behavioral Neuroscience	TSST	IGT	IGT performance	N.A.	B	40	0.15	Stress slows down learning, men ultimately do poorly, women do fine.	YES		N.A.	Larger
Starcke et al	2008	Behavioral neuroscience	~TSST	GDT	GDT net score	N.A.	B	40	0.15	Lower GDT score.	NO		No gender effect	Larger
Pabst et al	2013	Behavioral neuroscience	TSST	GDT	GDT net score	N.A.	B	126	0.39	Lower GDT score.			Large subject pool	Larger
Putman et al	2010	Psychopharmacology	Cortisol admin	Rogers gambles	P(gamble)	N.A.	W	29	0.34	Gambled more when p(lose) was high.			N.A.	Larger (sort of)
Pabst et al	2013	Frontiers in behavioral neuroscience	TSST	GDT (gain only/loss only)	GDT net score	N.A.	B	80	0.26	Fewer risky choices in the loss domain; no effect in the gain domain.	NO	X	GDT EVs better matched.	Larger for losses
Buckert et al	2014	Frontiers in Neuroscience	~TSST	Gamble/guaranteed lotteries	P(gamble)	Ambiguity, working memory	B	75	0.21	More risk seeking for gains, only in cortisol responders (N = 26).	NO	X	55 stress, 20 control; variable probabilities	Larger for gains
Van Den Bos et al	2009	Psychoneuroendocrinology	TSST	IGT	IGT performance	N.A.	B	33	0.14	Males worse, females better if mild, worse if strong cort.	YES		N.A.	Larger & Smaller
Lighthall et al	2009	PLoS One	CPT	BART	# balloon pumps	N.A.	B	45	0.17	Men pump more, women pump less.	YES		N.A.	Larger & Smaller
Pabst et al	2013	Behavioral brain research	TSST	GDT	GDT net score	N.A.	B	40	0.15	More risk averse 5&18min after, less risk averse 28min after.			All males; 10 subj/4 groups	Larger & Smaller
Von Helversen & Rieskamp	2013	Conf. Proceedings	CPT	Two gamble task	P(gamble)	N.A.	B	69	0.24	More risk with low outcome, less risk with high outcome gambles.	NO		No gender fx w stress	Larger & Smaller
Robinson et al	2014	PeerJ	Threat of shock	IGT	IGT performance	N.A.	W	47	0.52	Low anx/dep, more risk averse; high anx/dep, more risk seeking.			Interaction w/ trait anx & BDI scores	Larger & Smaller
Porcelli & Delgado	2009	Psychological Science	CPT	Two gamble task	P(gamble)	N.A.	W	27	0.32	More risky in losses, less risky in gains.		X	Single valence trial types	Smaller
Cingl & Cahlikova	2013	Discussion paper	TSST	Gain-only simple lottery questionnaire	Change point	N.A.	B	78	0.26	Lower certainty equiv.			Dropped inconsistent subj; men are p = 0.1, women are p = 0.14; correlation w/ cort is sig	Smaller
von Dawans et al	2012	Psychological science	TSST	Two gamble task	P(gamble)	N.A.	B	67	0.23	No change in risk aversion.			Also had sharing game, punishment game, trust game	No change
Lempert et al	2012	Frontiers in Psychology	~TSST	Gain-only lotteries	AUC of prob. discounting rate	Temporal discounting	B	113	0.36	No effect of stress on gambling; interaction btwn chronic & acute stress on temporal discounting.			All males; variable probabilities; staircasing procedure	No change
Delaney et al	2014	Discussion paper	CPT	Holt and Laury scale	Change point	Temporal discounting and probability weighting	W	90	0.80	No change in risk aversion			N.A.	No change
Kandasamy et al	2014	PNAS	Cortisol admin	Two gamble task	Curvature	Probability weighting	W	36	0.42	More risk averse with chronic cort (and no fx with acute cort).	NO		No gender fx; lotteries are complex	No change
Chumbley et al	2014	Psychological science	N.A. (Tonic cortisol)	DOSE (based on Sokol-Hessner et al, 2009 task)	Curvature	Loss aversion	W	53	0.57	No relationship of cort to rho; neg corr w/ Lambda.			Only fx w/ chronic!	No change
Sokol-Hessner et al			CPT	Sokol-Hessner et al, 2009 task	Curvature	Loss aversion, consistency	W	120	0.90	No effect of acute stress on risk att, loss aversion, or consistency.	NO		N.A.	No change
