# Supplementary material for: Acute stress does not affect risky monetary decision-making
Source: Neurobiol Stress. 2016 Nov 2;5:19–25. doi: 10.1016/j.ynstr.2016.10.003 (PMC5145911; doi:10.1016/j.ynstr.2016.10.003)
Supplement: Appendix [file mmc5.docx]

**Appendix: Supplementary Analyses & Discussions**

Sokol-Hessner, Raio, Gottesman, Lackovic, & Phelps, *Acute stress does not affect risky monetary decision-making.*

**1. Cortisol**

Participants could be divided into groups on the basis of which conditions they experienced (Stress and Control) on which days: Group 1 was Control/Control, Group 2 was Control/Stress, Group 3 was Stress/Control, and Group 4 was Stress/Stress.

The CPT consistently raised cortisol levels. In Group 2 and Group 3 (groups experiencing both the Stress and Control conditions), the change in cortisol relative to baseline at the third (pre-task) and fourth (post-task) time-points was significantly greater in the Stress condition than in the control condition. For Group 4 (Stress/Stress), there were weak differences in stress responses at the third (pre-task) time-point (paired t-test, p = 0.097), and significant differences at the fourth (post-task) time-point (p = 0.04).

Testing the third and fourth time-points against each other, there were no significant differences across all participants on Day 1 (p = 0.43), though there was a significant difference on Day 2 (p = 0.00002) with the fourth timepoint being less than the third.

A mixed effects regression on ΔCortisol values on both days (defined as log([Cortisol_3_-Cortisol_1_]+0.5)-log(0.5), as in the Methods) with random effects for the subject-level intercept, and fixed effects regressors representing day, stress condition, gender, and the two- and three-way interactions between day, condition, and gender found a significant intercept (p = 0.0003) and a significant effect of condition (p = 0.04), but no other significant effects (all p’s > 0.2).

See Figure S1 for plots of mean cortisol levels separated by group, timepoint, and day.

Baseline cortisol was weakly correlated with the session start time across participants on both Day 1 (Spearman’s Rho = -0.28, p = 0.002) and Day 2 (Spearman’s Rho = -0.17, p = 0.065), but the change in cortisol showed no such correlation on either Day 1 (Spearman’s Rho = 0.003, p = 0.97) or Day 2 (Spearman’s Rho = 0.01, p = 0.91).

There were no significant differences in baseline cortisol when collapsing across groups and testing Day 1 versus Day 2 (t(118) = 1.17, p = 0.25), nor when testing Day 1 versus Day 2 independently for each of the four groups (all p’s > 0.28).

CPT effects on the change in cortisol did not significantly differ between males and females. A mixed-effects linear regression in R (with lmer) predicting individuals’ change in cortisol at the 3^rd^ timepoint with a random intercept and fixed effects for Day, Stress, Gender, and all interactions found significant effects for the intercept (β = 0.06, p = 1.3 x 10^-8^) and Stress (β = 0.07, p = 8.9 x 10^-16^), but no main effects for Day, Gender, or any of the interaction terms (all p’s > 0.17).

**2. Additional hierarchical Bayesian models of decision-making**

In addition to the hierarchical Bayesian models of behavior mentioned in the main text, we ran two additional models to ensure that the lack of a finding for stress was not simply due to model complexity.

Model 3 was identical to Model 1 (modeling the effect of day and the stress condition), with the exception that hierarchical group- and individual-level terms were included to model the effect of the stress condition on risk attitudes (ρ) only (i.e. not for loss aversion [λ] or consistency [μ]). If the effect of stress on risk attitudes was weak, the extra degrees of freedom given to the effects of stress on loss aversion and consistency might have soaked up that variance, hiding the weak effect. However, the samples from Model 3 indicate that this was not the case, as the 95% CIs for the group mean effect of stress on risk attitudes still squarely spanned zero ([-0.05 0.05]).

Model 4 was identical to Model 2 (modeling the effect of day and the effects of parametric variation with ΔCortisol), save that the effect of ΔCortisol was modeled as single, fixed-effects terms (e.g. only one term was estimated for the change due to ΔCortisol in risk attitudes for all participants; one term was estimated for the change due to ΔCortisol in loss aversion for all participants, etc). Nevertheless, this model produced identical results as Model 2, with all 95% CIs spanning zero for the effect of ΔCortisol on risk attitudes ([-0.17 0.06]), loss aversion ([-0.22 0.32]), and consistency ([-0.29 0.39]).

**3. Nonhierarchical Maximum Likelihood Models**

In addition to using hierarchical Bayesian fitting procedures, we also used classic maximum likelihood estimation (MLE), exactly as implemented in previous studies with this task ([Sokol-Hessner et al. 2009](#_ENREF_6); [Sokol-Hessner et al. 2013](#_ENREF_4); [Sokol-Hessner et al. 2014](#_ENREF_5); [Sokol-Hessner et al. 2015](#_ENREF_7)). Though we believe the Bayesian approach superior to MLE, for consistency’s sake with prior literature, we additionally applied MLE procedures to our data and model.

Briefly, equations 1, 2, and 3 (from the main text) were used to compute a probability of selecting the risky option (given values for parameters ρ, λ, and μ) that was then matched with participants’ observed choices to compute the likelihood of the data given those parameters. This likelihood was then maximized (technically, the negative log likelihood was minimized) using interior-point algorithms as implemented in MATLAB’s “fmincon” function. This was done independently for each individual on each day (so each participant had two ρ values, two λ values, etc).

For all participants, MLE-fit parameters predicted participants’ behavior significantly better than chance (likelihood ratio tests of the full model against a null [chance] model; all p’s < 0.05).

Collapsing across the Stress/Control and Control/Stress groups (N = 60), paired t-tests of stress versus control revealed no systematic differences in ρ, λ, or μ (all p’s > 0.4).

Performing linear regressions on the parameter estimates with regressors for, Day, Condition, and Day x Condition revealed only trending effects of Day to increase λ (p = 0.1) and a significant effect of Day in increasing μ (p < 0.001) (the regression also included constants for each parameter, akin to a baseline value). An identical regression substituting ΔCortisol for Condition identified a significant effect of Day in increasing μ (p = 0.006) and a trending effect of ΔCortisol to increase μ (p = 0.1).

To look at our data from a between-subjects perspective, we eliminate half our data and only use that from Day 1 to compare the 60 Stress participants with the 60 Control participants. We note that this approach obviously greatly reduces our statistical power, and fails to take into account individual differences in decision-making as well as the two-day design with the Bayesian hierarchical modeling that we used in the main manuscript. Nevertheless, if we use the maximum likelihood estimates for behavior on Day 1 and two-sample t-tests, we find that there is no difference between Stress and Control for ρ (p = 0.92), λ (p = 0.35), or μ (p = 0.59).

As mentioned above and in the main text, we believe the Bayesian approach to be substantially superior to the MLE approach, as the Bayesian approach includes the (reasonable) assumption that our participants are similar to one another, and thus directly estimates the group-level parameters that here we must noisily and imperfectly infer.

**4. Curvature in the gain and loss domains**

One possible weakness of our design and analysis is that our task and model were not designed to allow the estimation of separate curvature coefficients (i.e. risk attitudes) for the gain domain and the loss domain. While it is well understood that the utility function is generally concave for gains and convex for losses (as modeled here and in many other places), the literature is generally equivocal as to whether the *degree* of curvature differs as a function of domain ([see page 661, Booij and van de Kuilen 2009](#_ENREF_1)). Additionally, while some studies have found that the effect of stress on risk taking differs in the gain and loss domains, their effects have been in opposite directions ([Porcelli and Delgado 2009](#_ENREF_3); [Pabst et al. 2013](#_ENREF_2)) (see Table S1).

Were stress to affect curvature in one domain but not the other, it’s still possible we would have picked up this change in our study as a small, subtle shift in risk attitudes (due to averaging over a change in curvature and a lack of a change in curvature), but we did not see any such effect in our model fits. Even if the effect was isolated to the loss domain, then a change in estimates of loss aversion might have been observed instead (which we also did not see). Thus, perhaps the only possibility we cannot reasonably reject is that stress affects risk attitudes in the gain and loss domains in opposite directions (e.g. reducing risk seeking over losses while increasing risk aversion over gains), thus canceling out any global, average effect on curvature. We think this unlikely, but again, cannot rule it out.

**5. Participant exclusion criteria**

We excluded any potential participants who reported possibly being pregnant, taking antidepressants or anti-anxiety medication, or having a history of heart problems or blood pressure problems. All participants also reported not eating or drinking anything except water for 1 hour prior to the beginning of the experiment.

**Captions**

**Table S1.** Brief summaries of 15 extant papers examining the effect of acute stress on risky decision-making. This set of papers is meant to be representative, not necessarily exhaustive. Under “Stressor”, TSST = Trier Social Stress Test; CPT = Cold Pressor Test. Under “Task”, IGT = Iowa Gambling Task; GDT = Game of Dice Task; Rogers gambles = a gamble set from Rogers et al 2004; BART = Balloon Analogue Risk Task. To facilitate the comparison of studies, the column identified as “Pwr” illustrates the power of that study to detect a theoretical effect size of 0.3 (a small-to-medium size effect) as calculated by MATLAB’s sampsizepwr function. For each study, the simplified power calculation assumed either a two-sample t-test (between-subjects) or a paired-sample t-test (within-subjects), with two-tailed α = 0.05. For between-subjects designs, the number of participants in each group was assumed to be half the total N, rounded up (actual group sizes were used for Buckert et al as their groups were different in size by a factor of 2.75). The power value for the current study is a simplified estimate – this study contained both between- and within-subjects elements that informed each other, as well as a more powerful and robust estimation procedure. The column identified as “M/F?” identifies studies that tested for gender effects, and if they did, whether they found them. Studies that tested for gender received either a “YES” or “NO” (to see the exact effect, see “Rough Finding” and “Notes”), whereas those that did not are left blank. The column identified as “G/L?” identifies studies that looked for effects separately in the gain and loss domains; if they did so, they received an “X”, otherwise were left blank. The column identified as “Implied Effect on ρ” summarizes the finding in terms of the effects of acute stress on utility curvature as represented by the parameter ρ. It is commonly found that individuals are risk averse in the gain domain and risk seeking in the loss domain. If studies found more risk aversion for gains and/or more risk seeking for losses, that is consistent with a smaller ρ; less risk aversion for gains and less risk seeking for losses is consistent with a larger ρ. Papers are sorted by main finding and then year (within main finding). Chumbley et al (2014) is listed separately, as they examined chronic levels of cortisol, not acute stress. The current paper is included at the very bottom of the table, for comparison’s sake.

**Figure S1.** Changes in cortisol as a function of day and condition. Each graph depicts the average change in cortisol (ug/ml) for one group of participants (N = 30) on Day 1 and on Day 2 (solid and dashed lines respectively). The Control condition is indicated in blue, Stress in red. Error bars are standard errors of the mean.

**Figure S2.** Probability of gambling as a function of day and group. Each graph depicts the raw probability of gambling across all trials on Day 1 (x-axis) and Day 2 (y-axis) for a given group. Groups are indicated by the “C” and “S” squares in the upper left hand corner of each graph; the first square indicates the condition on Day 1, and the 2^nd^ square the condition on Day 2. Blue squares with “C” indicate the control condition, while red squares with “S” indicate the stress condition. Each circle is one participant, and the large circle in each graph is the group mean.

**Figure S3**. Changes in decision-making due to Cortisol and Day (Model 2). Group mean changes in each of risk attitudes (ρ, green), loss aversion (λ, red), and consistency (μ, blue) due to repeated participation (“Day”) or due to parametric changes in cortisol (“Cortisol”). Each histogram represents 12,000 samples from Model 2 (see Methods). 95% Confidence intervals are indicated for each histogram with dashed lines. Intervals excluded zero only for changes in loss aversion and consistency due to Day.

References

Booij, A. S. and G. van de Kuilen (2009). "A parameter-free analysis of the utility of money for the general population under prospect theory." Journal of Economic Psychology **30**(4): 651-666.

Pabst, S., M. Brand, et al. (2013). "Stress effects on framed decisions: there are differences for gains and losses." Frontiers in Behavioral Neuroscience **7**: 1-10.

Porcelli, A. J. and M. R. Delgado (2009). "Acute Stress Modulates Risk Taking in Financial Decision Making." Psychological Science **20**(3): 278-283.

Sokol-Hessner, P., C. F. Camerer, et al. (2013). "Emotion Regulation Reduces Loss Aversion and Decreases Amygdala Responses to Losses." Social Cognitive and Affective Neuroscience **8**: 341-350.

Sokol-Hessner, P., C. A. Hartley, et al. (2014). "Interoceptive ability predicts aversion to losses." Cognition & Emotion.

Sokol-Hessner, P., M. Hsu, et al. (2009). "Thinking like a trader selectively reduces individuals' loss aversion." PNAS **106**(13): 5035-5040.

Sokol-Hessner, P., S. F. Lackovic, et al. (2015). "Determinants of propranolol's selective effect on loss aversion." Psychological Science **26**(7): 1123-1130.
